# Supplementary material for: Spatial constraints govern competition of mutant clones in human epidermis
Source: Nat Commun. 2017 Oct 24;8:1119. doi: 10.1038/s41467-017-00993-8 (PMC5654977; doi:10.1038/s41467-017-00993-8)
Supplement: Supplementary file 1 — Supplementary Information [file 41467_2017_993_MOESM1_ESM.pdf]

Supplementary Methods.

A full description of the lattice model is provided in *Methods*, here we provide additional detail to that discussed in the main text.

Neutral drift. For neutral mutations, the computational model is equivalent to a model of neutral drift and has only two parameters: $r\lambda$ the rate of stem cell loss / replacement in the basal layer and $r\omega$ the rate of neutral mutation. Consistent with the predictions of a mathematical model of neutral drift[1], a plot of the 1st incomplete moment of the clone size distribution shows an excellent fit to $e^{-(n-n_0)/r\lambda t}$ (Supplementary Figure 5a-h).

Mathematical modelling also indicates that the shape of the clone size distribution when plotted against the 1st incomplete moment is independent of $r\omega$ the genomic mutation rate[1]. To investigate this in our simulation, we simulated clonal evolution for 3 years. This shows the shape of the clone size distribution and the maximum size of clones to be independent of mutation rate across three orders of magnitude from $10^{-1} - 10^{-3}$ mutations cell^{-1}day^{-1}. When the incidence of mutations was reduced to 10^{-4} cell^{-1}day^{-1} although the shape of the clone size distribution remains unchanged, the maximum size of mutation detected was reduced. Conceptually this is easy to understand since a very low incidence of mutation will extend the time taken for the first mutations to begin expansion and therefore there will be less time available for mutations to increase in size. Having confirmed that the rate of neutral mutation $r\omega$ does not influence the findings of the model we employ a rate of 10^{-3} cell^{-1}day^{-1} for the remainder of the simulations.

Effects of cellular migration on neutral drift. The above simulation of neutral drift assumes that the rate of cellular migration is essentially negligible within the basal layer of the epidermis. Whilst keratinocytes migrate in culture and during wound healing *in vivo* indirect evidence suggests that rates of cellular migration in the steady state basal layer of the epidermis are likely to be low: Confluent sheets cultured keratinocytes do not move relative to one another, for example in our experiments with GFP labelled cell clones[4,5]. Furthermore, lineage tracing of healing mouse wounds show sheets of clonally related cells moving in concert, rather than as single cells[6].

Both staining for *TP53*-mutant patches in the human epidermis[7,8] and lineage tracing of mutant Notch clones in the mouse eosophagus[3] or Hedghog singalling in the mouse epidermis[9] demonstrates that clones expand as spatially contiguous clusters rather than scattered, dispersed cells. To investigate the consequences of cellular migration for the spatial distribution of clones, we varied the rate of cellular migration in the lattice. Cellular migration was modelled by stochastically swapping the position of adjacent cells on the lattice. It can be seen that the shape of the observed clone size distribution is independent of the rate of cellular migration (Supplementary Figure 6a,c,e,g,i), however the spatial distribution of mutant clones changes as the rate of migration increases: At low rates of cellular migration (Supplementary Figure 6b,d) clones are cohesive, at higher rate of cellular migration (Supplementary Figure 6h,j) clones are instead diffused and scattered. This implies that *in vivo* rates of cellular migration are essentially negligible relative to the time required for cellular division. The diameter of a stem cell in the epidermal basal layer is approximately 10-15 $10\,\mu m$[10] therefore the highest rate of cellular migration modelled is a minimum of $10\,\mu m$/day.

Non-neutral dynamics. In order to give rise to a competitive advantage, a mutation must alter the dynamics of stem cell replication or loss within the basal layer of the epidermis. To examine the consequences of these non-neutral mutations for clone size distributions, we modelled the stochastic occurrence of a two-fold reduction in the rate of loss from the stem cell compartment for mutant cells (Supplementary Figure 7a-x). Thus $r\lambda$, the rate of loss/replacement in wild type cells, is 0.5 cell^{-1}week^{-1}and $r\phi$, the rate of loss/replacement for mutant cells is 0.25 cell^{-1}week^{-1}. Clones above the size threshold that would be detected by high throughput sequencing consistently exhibit the anticipated inverse exponential size distribution (Supplementary Figure 8a-l) and as expected this is not dependent upon the non-neutral mutation rate. The distribution of very small clones reflects neutral mutations arising in the remaining wild type cells and within primary clones that are subject to neutral drift.

It is probable that the rate of neutral mutations greatly exceeds the rate of non-neutral mutations in vivo, although the proposed mechanism does not require this (Supplementary Figure 9). Where non-neutral mutations are more frequent the maximum clone size achieved is reduced, since expanding primary clones collide with one another reverting to neutral drift. This is observed when the model is run with a 10-fold higher non-neutral mutation rate (Supplementary Figure 9). Thus, paradoxically a higher non-neutral mutation rate gives rise to a smaller clone size distribution (Supplementary Figure 7q,r) reflecting collision between expanding mutant clones on the lattice. For low rates of non-neutral mutation, (Supplementary Figure 7u,v,w,x) a large excess of small clones is noted. However when the larger clones that are detectable by high throughput sequencing are plotted separately (Supplementary Figure 8b,c,e,f,h,i,k,l) the shape of the distribution formed by

the larger clones is clear.

Nucleation of secondary clones at the boundary. The dynamics of clonal splitting are best appreciated by observing dynamic evolution of the lattice (Supplementary Video 1). In the initial stages of the simulation all of the stem cells are wild type (white, not visible). Subsequently rare clones arise all of which (B_m) carry an identical non-neutral mutation that results in a 2-fold reduction in loss from the stem cell compartment. All cells carrying this non-neutral mutation are colored; the colour of the clone is an arbitrary function of the clonal label m. As the simulation progresses, these mutant clones acquire subsequent secondary mutations that change the clonal label and this is observed as the development of patches of another colour within the main colonies. When a neutral mutation arises within the body of a clone it does not expand to a large extent, however when a neutral mutation arises at the periphery of an expanding clone it can expand to form a large segment of another colour. The rate of non-neutral mutations in this simulation is (10^{-6} cell^{-1}day^{-1}).

Effects of magnitude of competitive advantage on non-neutral dynamics. The above simulation assumes that a non-neutral mutation leads to a 2-fold (50%) reduction in the probability of loss from the stem cell compartment. The precise magnitude of this effect is unknown, indeed it is likely that different non-neutral mutations will exhibit varying magnitudes of effect. Intuitively it seems likely that the magnitude of competitive advantage will not determine the shape of the clone size distribution, however we wished to investigate this by simulation. The simulation was therefore run for differing values of survival advantage. Where the survival advantage is 10% for

non-neutral mutations, there is no significant clonal expansion (Supplementary Figure 10a). However where the survival advantage for non-neutral mutations is greater than this (Supplementary Figure 10b-f) there is expansion of non-neutral clones with the anticipated clone size distribution. This indicates that the magnitude of the competitive advantage has little impact on the clone size distribution and therefore we assume a 2-fold advantage for subsequent simulations.

Effects of rate of cellular migration on non-neutral dynamics. As discussed above, the rate of cellular migration of stem cells within the basal layer of the epidermis is likely to be negligible relative to the time required for cellular division *in vivo*. Nevertheless, we wished to examine whether a high rate of cellular migration would be compatible with the proposed mechanism for clonal expansion of non-neutral mutations (Supplementary Figure 10g-k). The simulation was run with average rates of cellular migration varying from 0 (g) 10^{-3} cell^{-1}day^{-1} (h) 10^{-2} cell^{-1}day^{-1} (i) 10^{-1} cell^{-1}day^{-1} (j) and 1.0 cell^{-1}day^{-1}. For all rates of migration up to 1.0 cell^{-1}day^{-1} there is a good fit to the observed clone size distribution. For a rate of 1.0 cell^{-1}day^{-1} (Supplementary Figure 10k) there is a small excess of the smallest clones that reflects a modest increase in the maximum clone size achieved by neutral drift where there is a high rate of cellular migration. Therefore, a negligible rate of cellular migration is not required for the proposed model.

Simulation of a heterogenous stem cell compartment. Thus far we have assumed that the basal layer of the epidermis is a homogenous population of stem cells. However the human epidermal basal layer is in fact comprised of islands of stem cells that express high levels of β1- and α6-integrin surrounded by a 'sea' of more differentiated transit amplifying (TA) cells expressing

markers such as keratin 10 (Fig 4a-c). β1-integrin high cells are enriched in cells with higher proliferative potential[11]. We wished to investigate whether the initial spatial configuration of stem cells relative to transit amplifying cells alters observed clone size distributions (Online methods).

In keeping with experimental data[7], we assumed that stem cells had negligible rates of migration giving rise to coherent cell clusters whilst TA cells had higher rates of migration that give rise to dispersed clones. The location of stem cell clusters corresponds to the top of rete ridges (Fig 4a-c). These stem cell clusters are surrounded by TA cells and where stem cells leave these clusters they differentiate to TA cells. Stem cells self-associate such that TA cells are not observed to admix with the stem cell clusters (Fig 4a-c). This tendency of stem and TA cells to self-associate was modelled by introducing a lattice energy function into the simulation (Online methods). In accordance with whole mount staining of the basal layer of the epidermis (Figure 4a-c) stem cell clusters were large in comparison to the size of intervening regions containing transit amplifying cells (Figure 4d-g; Supplementary Video 2). The rate of cell loss / replacement was the same for both stem and TA cells. Simulations revealed that the maximum number of replications completed by a TA cell (Supplementary Figure 11a-l) and the rate of cellular migration of TA cells (Supplementary Figure 11m-v) determine the extent which TA cells spread from stem cell clusters to occupy intervening regions of the lattice.

In creating this model, our primary goal was to investigate the consequences of the initial spatial configuration of stem cells for observed clone size distributions. We have sought to accurately capture our current understanding of epidermal stem cell biology, however a number of parameters,

such as the number of divisions completed by TA cells or the rate of cellular migration of TA cells have not been experimentally determined. Fortunately, for the purposes of our model, these factors are likely of little consequence, since we have showed that the large clone size distribution primarily reflects those mutations that arise in expanding clones containing non-neutral mutations. From the perspective of these expanding clones, the identity and dynamics of cells occupying the regions into which they expand is of little consequence and for this reason the previous assumption of a homogenous compartment is likely reasonable.

Effects of stem cell cluster size in a heterogenous stem cell compartment. Whole mount staining of the basal layer of the human epidermis (Fig 4a-c) suggests that clusters of stem cells are large in comparison to the size of interspersed transit amplifying cells. However, we wished to explore whether a different initial configuration with smaller stem cell cluster sizes altered clone size distributions. We simulated clusters of stem cells with average diameter of 10 cells and average inter-cluster spacing of 20 cells (Sup Fig 12a-d) for 1000 days. In contrast to the simulation presented in Figure 4 where larger clusters of stem cells are stable even where the rates of stem cell loss / replacement are the same in stem and TA cells, for the smaller clusters in this simulation, it was found that stem cell clusters were no longer stable and are stochastically replaced by surrounding TA cells as a consequence of neutral drift. In order to assure persistence of these smaller stem cell clusters it was necessary to reduce the rate of cell loss from stem cell compartment in comparison to the TA compartment; a 2-fold reduction was sufficient. Stages in the evolution of this model in the presence of both neutral and non-neutral mutations are illustrated (Supplementary Figure 12e-h). Interestingly, in the absence of non-neutral mutations, there is a substantial

deviation of the clone size distribution from the predictions of neutral drift (Supplementary Figure 12i). However where non-neutral mutations are introduced (Figure 12j) the experimentally observed distribution is restored. Interestingly this was not the case for the larger stem cell cluster size and may reflect the presence of populations with different rates of stem cell loss / replacement or a consequence of the larger fraction of the compartment occupied by non-stem cells.

Effects of relaxation of spatial constraints. The models presented above, have assumed that each position on the lattice — which corresponds to a unique spatial location on the epidermal basement membrane — is occupied by a maximum of one cell. It is possible that this restriction is more extreme than *in vivo*. For example, cells may be compressed or become stacked upon one another effectively permitting more than one cell to occupy the same lattice position.

We wished to investigate whether a relaxation of this restriction alters the observed clone size distribution. The simulation of expansion of non-neutral mutations was modified to permit multiple cells at each lattice point (Supplementary Figure 13). Where multiple cells were located at the same lattice point, all were considered spatially equivalent for example having equal probability of undergoing replication to occupy an adjacent vacancy in the lattice. Clonal evolution was simulated on a 200$\times$200 lattice for a period of time equivalent to 10 years. A large number of cells in each lattice point is biologically implausible and therefore a maximum 6 cells per lattice point were simulated.

For comparison the clone size distribution arising from a single cell per lattice point is shown (Supplementary Figure 13a). It can be seen that a similar distribution is preserved for all cell

numbers up to 6 cells per lattice point (Supplementary Figure 13b-f), however the maximum size of the distribution achieved increases as the number of cells per lattice point increases. This indicates that the conclusions of our modelling are not dependent upon strict assumptions with regard to the occupancy of lattice positions.

Finally, in order to examine the consequences of cellular migration in combination with a relaxation of spatial constraints we performed two simulations — the first with a low rate of migration (0.5 cell diameters^{-1}day^{-1}) and a small reduction in lattice spatial occupancy stringency (up to 2 cells per lattice position) and the second with a high rate of cellular migration (0.5 cell diameters^{-1}day^{-1}) in combination with a marked relaxation of lattice spatial occupancy rules (up to 5 cells per lattice position). Whilst the former did not lead to a significant change in the shape of the clone size distribution (Supplementary Figure 13g), the latter led to a marked deviation (Supplementary Figure 13h). This is not unexpected since, as discussed above, a high rate of migration in combination with a large number of cells at each position in the basal membrane is biologically implausible.

a

Age	Tumour type, Site	Comorbidities
33	BCC, Right nasal ala	
61	BCC, Right temporal scalp	Multiple previous non-melanoma skin cancers
68	BCC, Mid forehead	Pityriasis rubra pilaris
73	Bowen's disease, Right temple	Previous non-melanoma skin cancer
76	BCC, Left nasal sidewall	
77	BCC, Right temple	
81	BCC, Right lateral neck	
82	BCC, Left cheek	
87	BCC, Left nasal sidewall	
87	BCC, Central forehead	

b

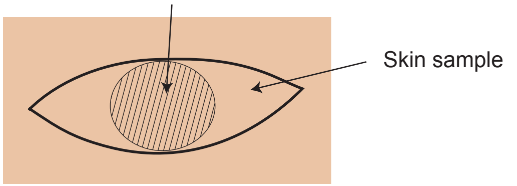

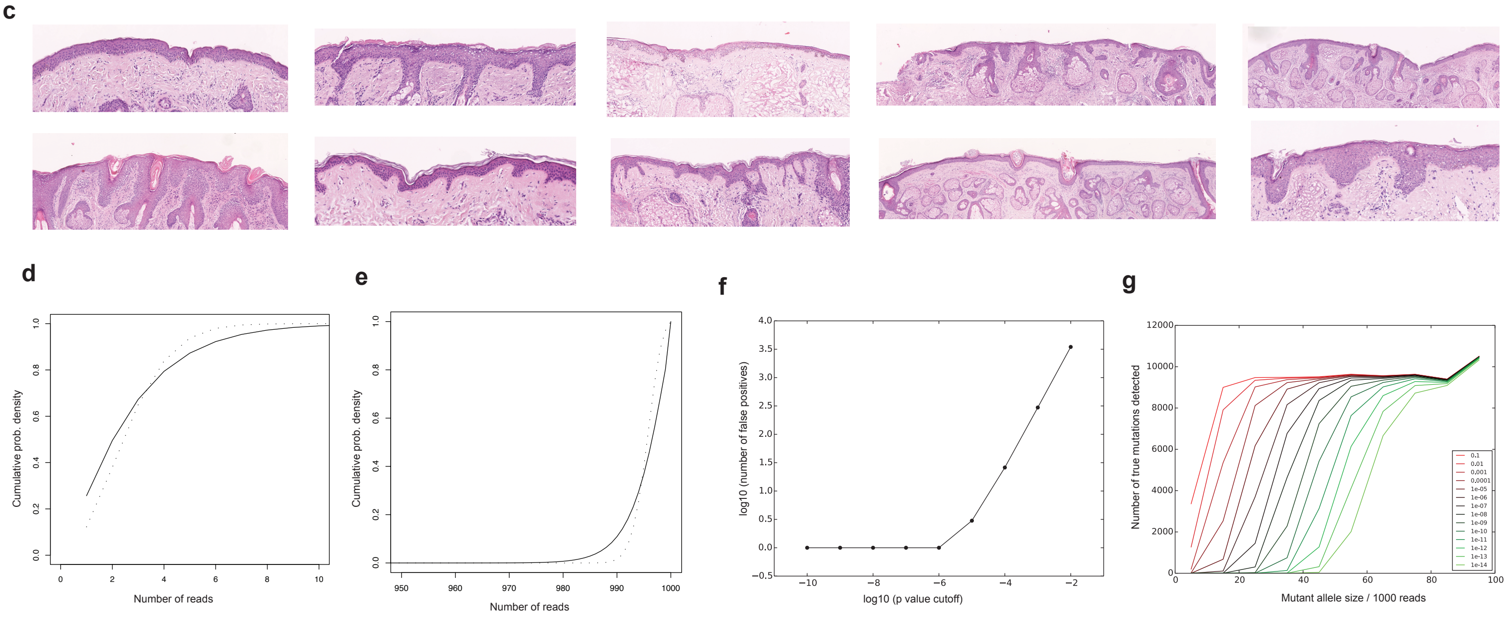

Supplementary Figure 1.
Ultradeep sequencing of skin cancer associated genes in preneoplastic epidermis.
a) Demographics, donor site and comorbidities for the patients from which skin samples were obtained. Two of the patients had a previous history of non-melanoma skin cancer and 1 patient had a diagnosis of Pityriasis rubra pilaris, an inflammatory dermatosis.
b) Excess skin sample was obtained from patients undergoing Mohs micrographic surgery for treatment of non-melanoma skin cancer lesions affecting the head and neck. Following removal of the skin tumour and confirmation that all margins are clear of residual disease the defect is reconstructed. Excess skin removed during the reconstruction was obtained.
c) H&E stained specimens from the 10 patients.
d) Fit of read density to beta binomial distribution (solid line) versus binomial distribution (dotted line) for background reads.
e) Fit of read density to beta binomial distribution (solid line) versus binomial distribution (dotted line) for wild type bases.
f) False discovery rate on simulated data according to p value (log 10 scale).
g) Sensitivity (true discovery rate) on simulated data according to p value.

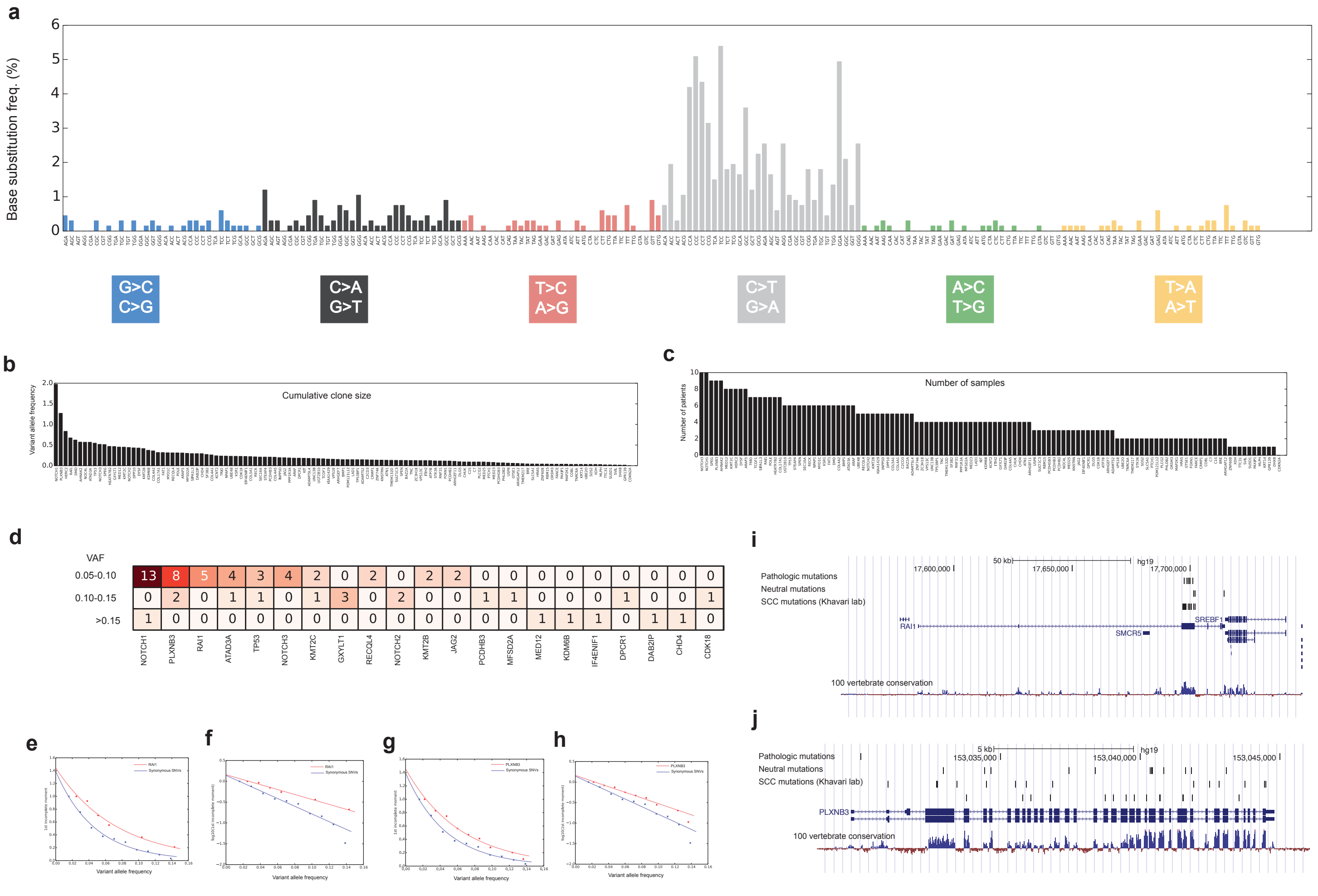

Supplementary Figure 2.

Mutations present in preneoplastic epidermis.

a) Base substitutions plotted according to trinucleotide context on the coding strand.

b) Cumulative size of mutations identified across all patients (VAF, variant allele fraction).

c) Number of patients (samples) for which a mutation was detected in each gene (maximum 10).

d) Identity of mutations identified in large clones.

e-f) Clone size distribution plotted as 1st incomplete moment for *RAI1* (red) compared to synonomous SNVs (blue) on a log (e) and linear (f) scale.

g-h) Clone size distribution plotted as 1st incomplete moment for *PLXNB3* (red) compared to synonomous SNVs (blue) on a log (g) and linear (h) scale.

i-j) Location of pathologic and neutral mutations in preneoplastic epidermis for (i) *RAI1* and (j) *PLXNB3*. The location of mutations in cutaneous SCC (Khavari lab data) is shown for comparison.

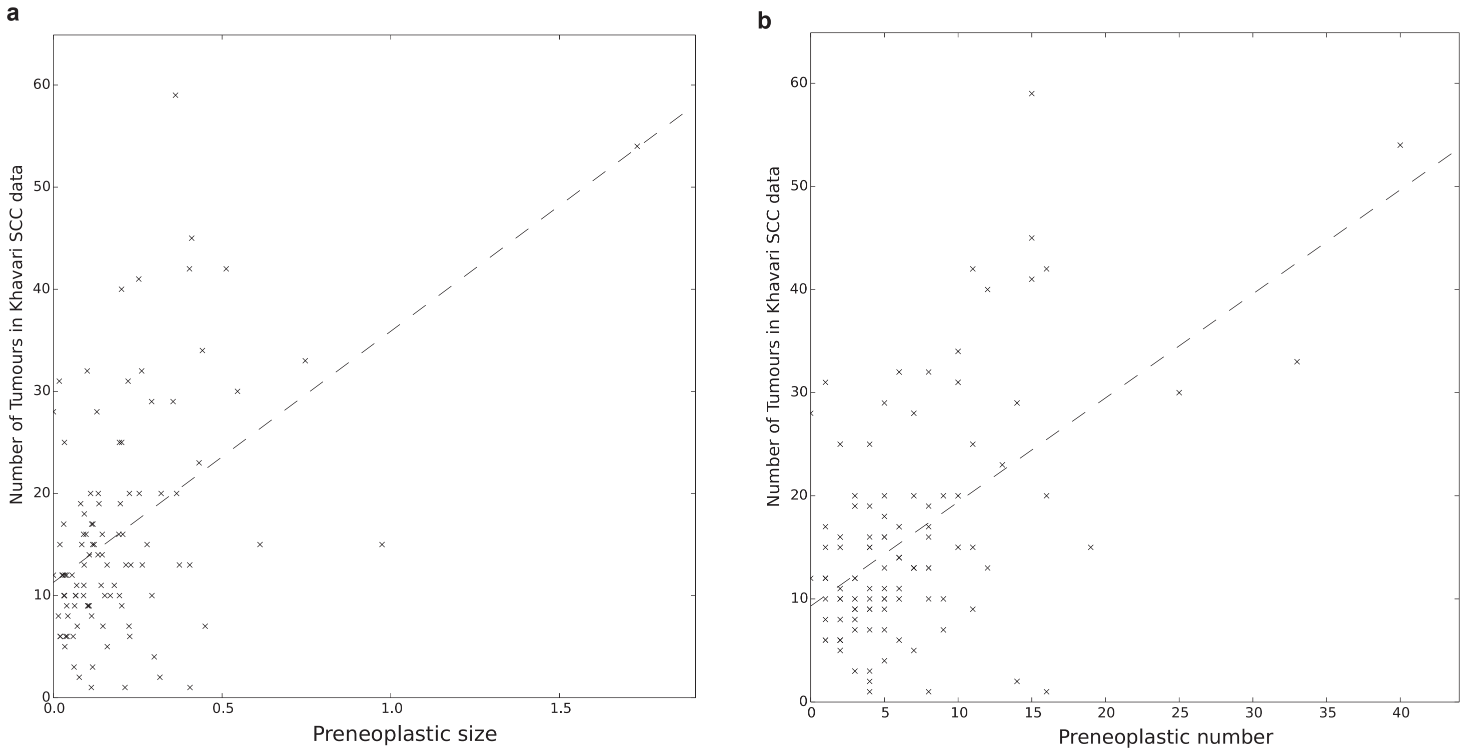

Supplementary Figure 3.

Comparison of mutations in preneoplastic epidermis and cutaneous SCC.

a) Comparison of incidence of mutation in cutaneous SCC (number of tumours) with cumulative clone size in preneoplastic epidermis (variant allele fraction). Pearsons correlation coefficient: R = 0.48, p = 1.4×10^{-7}.

b) Comparison of incidence of mutation in cutaneous SCC (number of tumours) with total number of mutations identified in preneoplastic epidermis. Pearson's correlation coefficient: R = 0.55, p = 6.9×10^{-10}.

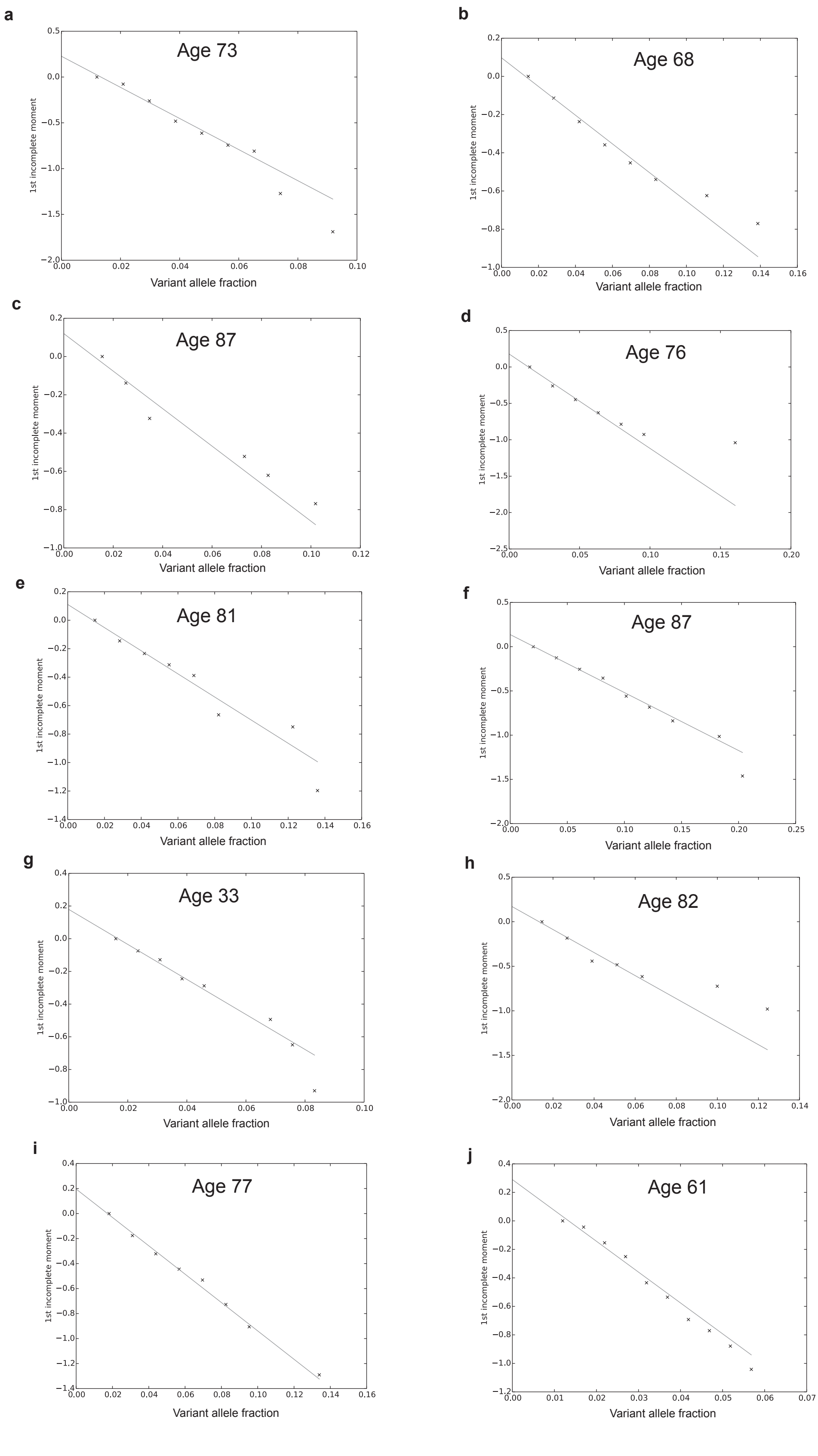

Supplementary Figure 4.

Per-sample mutant clone size distributions.

a-j) Mutant clone size distributions plotted against 1st incomplete moment for each sample on a log scale. Each sample is from a separate patient. The age of each patient is indicated.

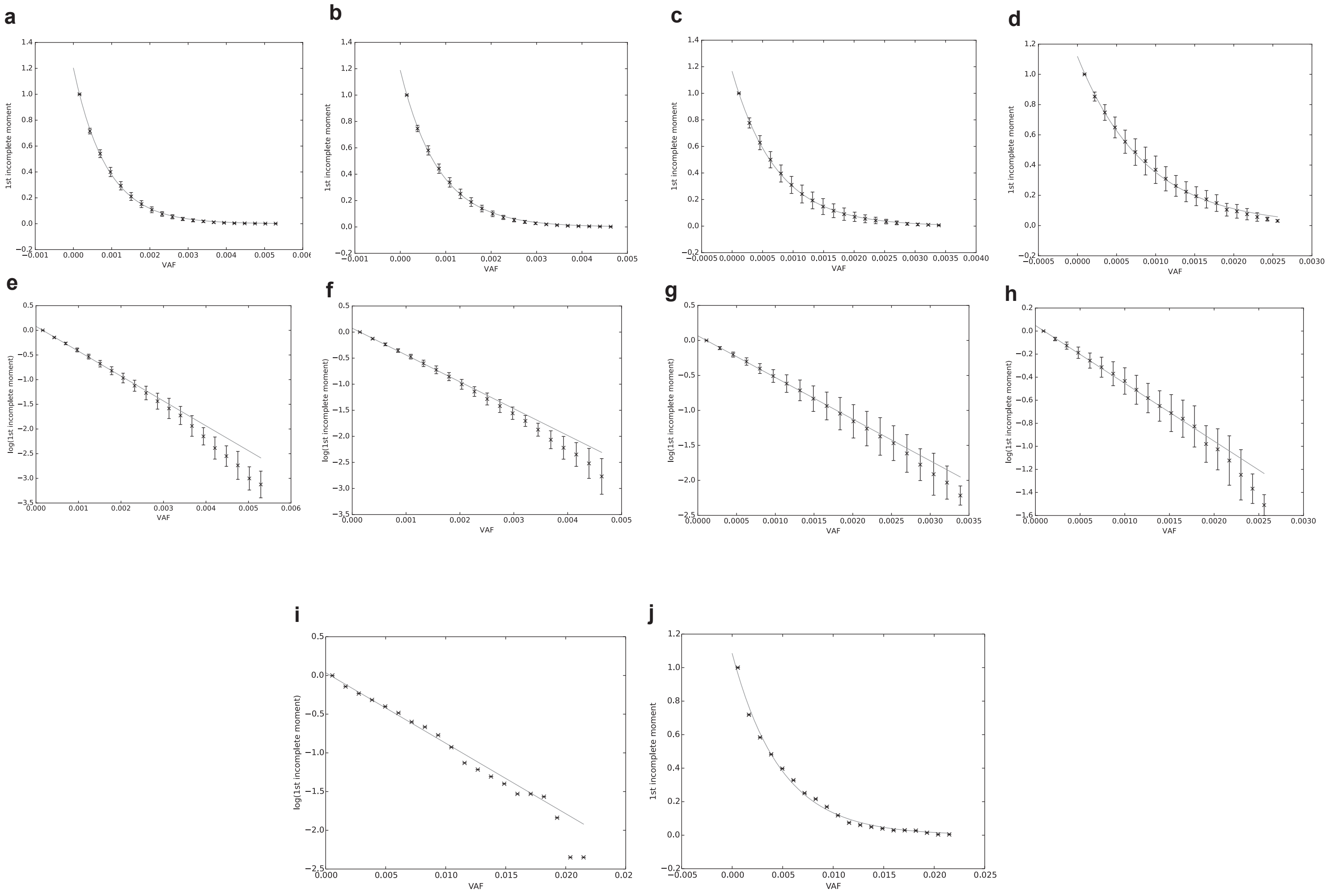

Supplementary Figure 5.

Neutral drift arising from simulation of stem cell evolution on a hexagonal lattice.

a-h) Clone size distributions on linear (a-d) and log (e-h) scales resulting from simulation of stem cell clonal evolution on a 200x200 hexagonal lattice for a period of simulation equal to 3 years. The rate of stem cell loss/replacement is 0.5 $cell^{-1}week^{-1}$. All stem cells have equal replicative potential and accrue neutral mutations stochastically. The rate of neutral mutation is 0.1 $cell^{-1}day^{-1}$ (a,e), 0.01 (b,f), 0.001 (c,g) and 0.0001 (d,h). Each simulation was repeated 10 times with different random seeding and mean +/- S.D. is displayed.

i-j) 100 years simulation of neutral drift in a 400x400 lattice equivalent to a $16mm^2$ region of the basal layer of the epidermis. A rate of stem cell loss/replacement of 0.5 $cell^{-1}week^{-1}$ was assumed consistent with experimental evidence.

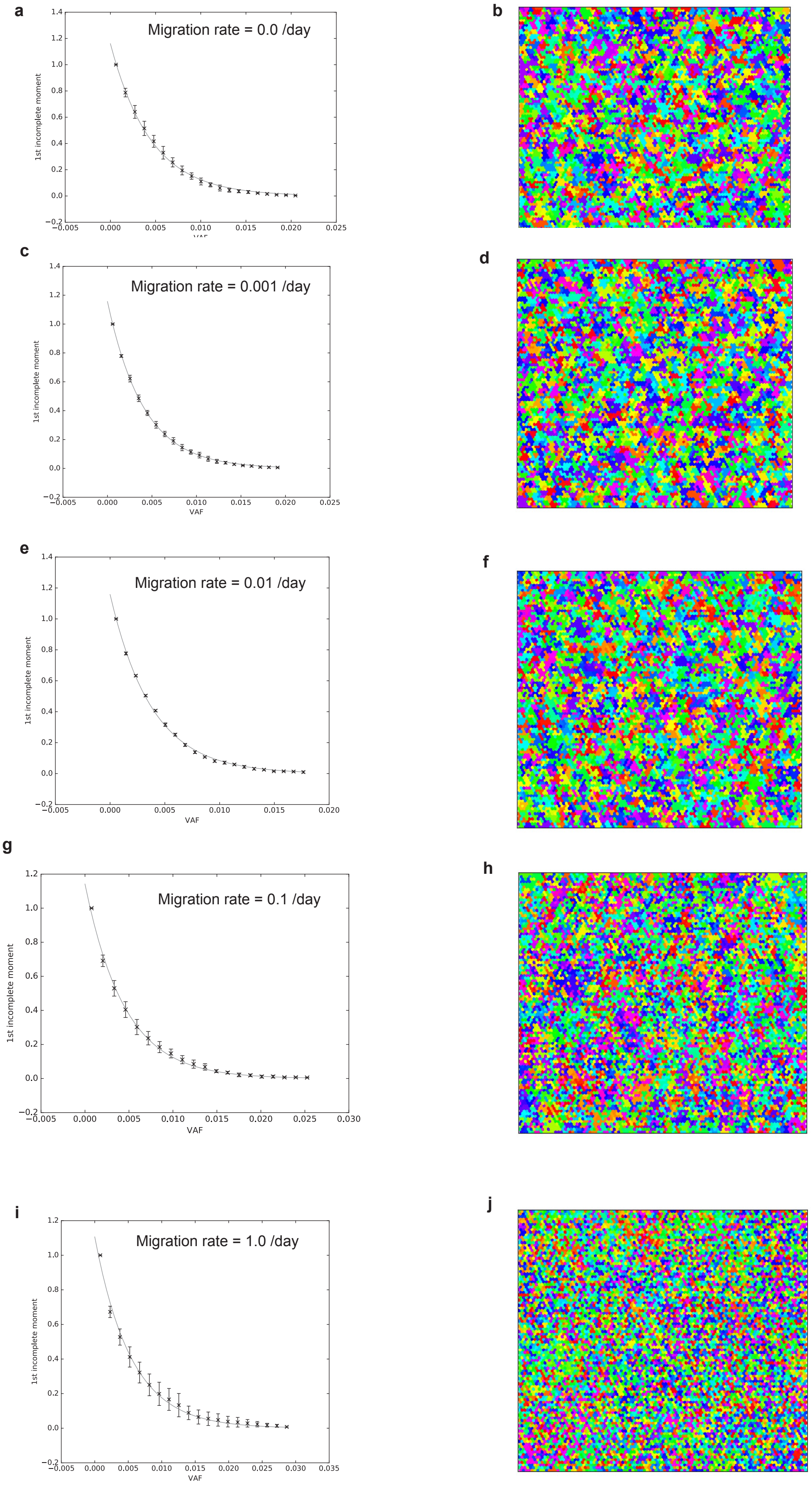

Supplementary Figure 6.
Effects of cellular migration rate on clone size distribution and spatial architecture of clones arising by neutral drift within the lattice. Clone size distributions (a,c,e,g,i) and the spatial distribution of clones (b,d,f,h,j; each clone is labeled with arbitrary color) after 1000 days of simulation on a 100x100 lattice. The average rate of cellular migration in the lattice was varied from 0.0 cell^{-1}day^{-1} (a,b) 10^{-3} cell $^{-1}$day^{-1} (c,d) 10^{-2} cell^{-1}day^{-1} (e,f) 10^{-1} cell^{-1}day^{-1} (g,h) 1.0 cell^{-1}day^{-1} (i,j). Neutral mutation rate was 10^{-2} cell $^{-1}$day^{-1}. Each simulation was repeated 3 times with different random seeding and mean +/- S.D. is displayed.

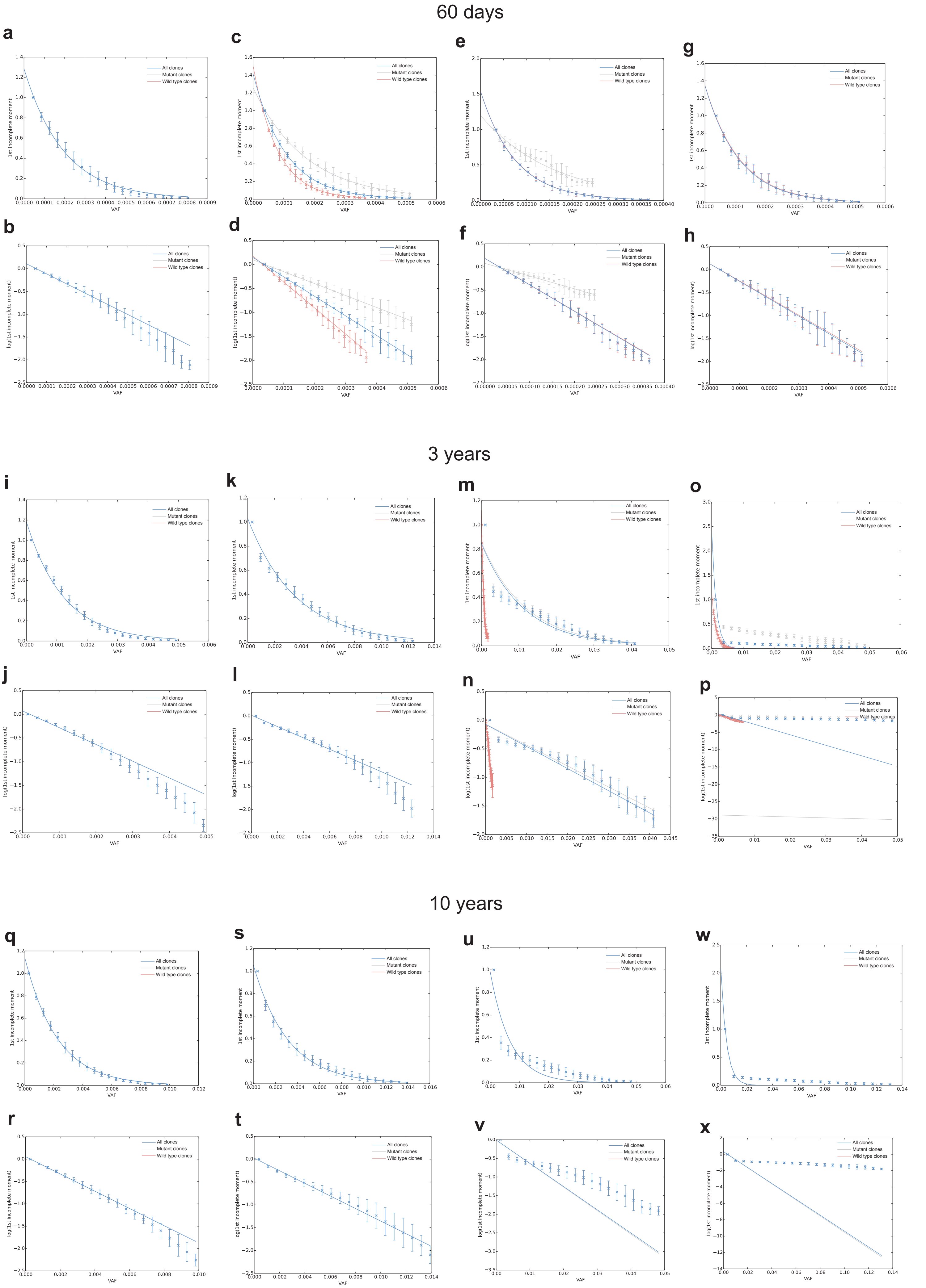

Supplementary Figure 7

Supplementary Figure 7.

The effects of non-neutral mutation on clone size distributions in a simulation of stem cell evolution on a hexagonal lattice. Cells were simulated on a 200x200 hexagonal lattice with a neutral mutation rate of 10^{-2} for periods of simulation equivalent to 60 days (a-h), 3 years (i-p) and 10 years (q-x). The rate of stem cell loss/replacement was 0.5 $cell^{-1}week^{-1}$. Non-neutral mutations which reduced by half the probability of loss from the stem cell compartment were allowed to arise stochastically with rate 10^{-2} $cell^{-1}day^{-1}$ (a,b,i,j,q,r); 10^{-3} (c,d,k,i,s,t); 10^{-4} (e,f,m,n,u,v) and 10^{-5} (g,h,o,p,w,x). The size distribution for clones with VAF$>$0.007 are plotted against 1st incomplete moment separately for all clones (blue), wild type/neutral mutations (red) and clones carrying a non-neutral mutation (grey). Each simulation was repeated 10 times with different random seeding and mean +/- is displayed.

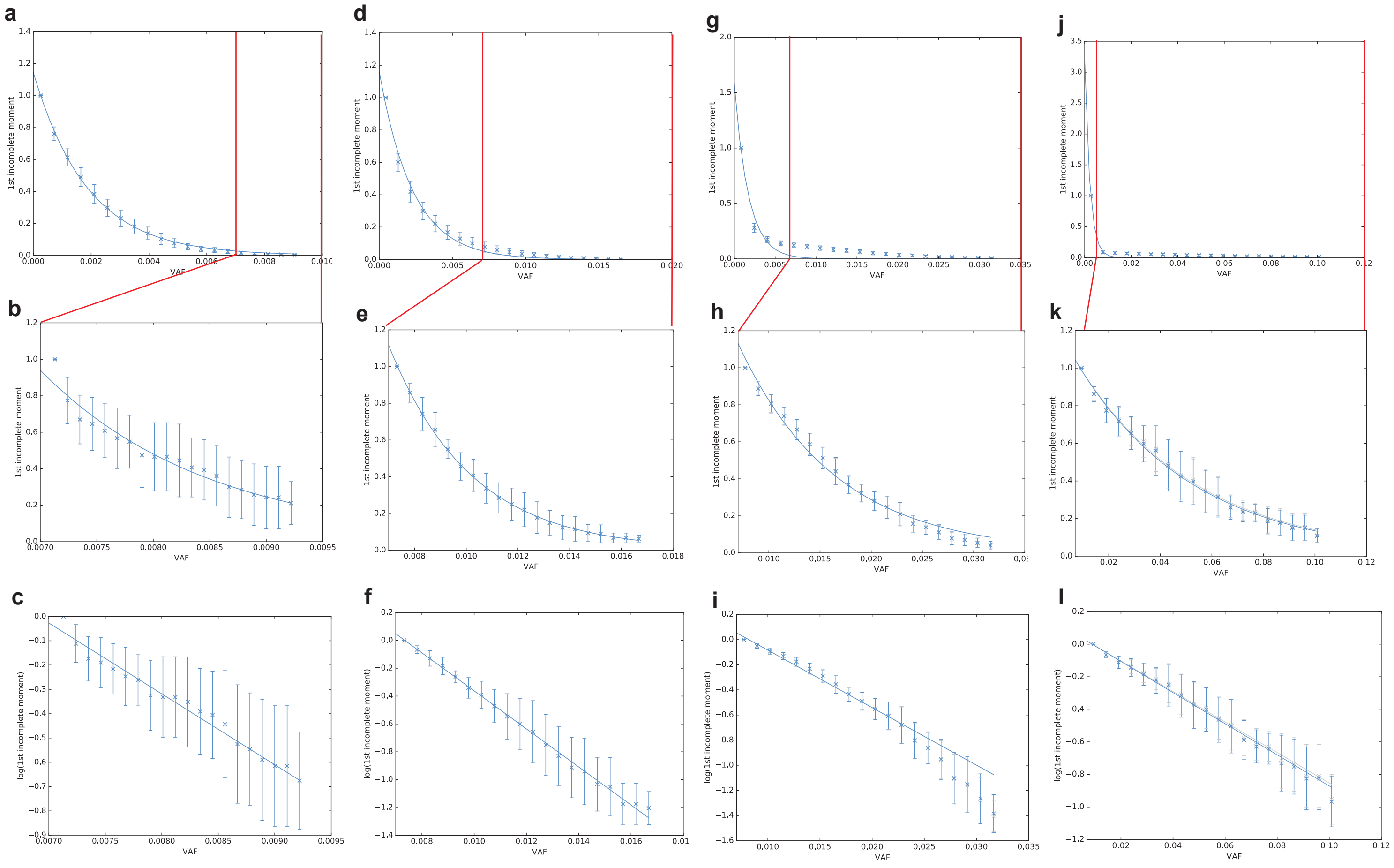

Supplementary Figure 8.
Clones detectable by high throughput sequencing. The region of the clone size distribution with a VAF larger than the (the smallest clones detected in our experiment) is plotted. Cells are simulated on a 200x200 hexagonal lattice for the equivalent of 10 years with a neutral mutation rate 10^{-3} with rate of stem cell loss/replacement 0.5 $cell^{-1}week^{-1}$. Non-neutral mutations which reduced by half the probability of loss from the stem cell compartment were allowed to arise stochastically with rate 10^{-3} $cell^{-1}day^{-1}$ (a-c); 10^{-4} (d-e); 10^{-5} (g-i) and 10^{-6} (j-l). Clone size distributions are plotted against 1st incomplete moment for all clones (a,d,g,j) and clones with VAF > 0.007 (b,e,h,k,c,f,i,l) on linear (b,e,h,k) and log (c,f,i,l) scales. Each simulation was repeated 10 times with random seeding and mean +/- S.D. is displayed.

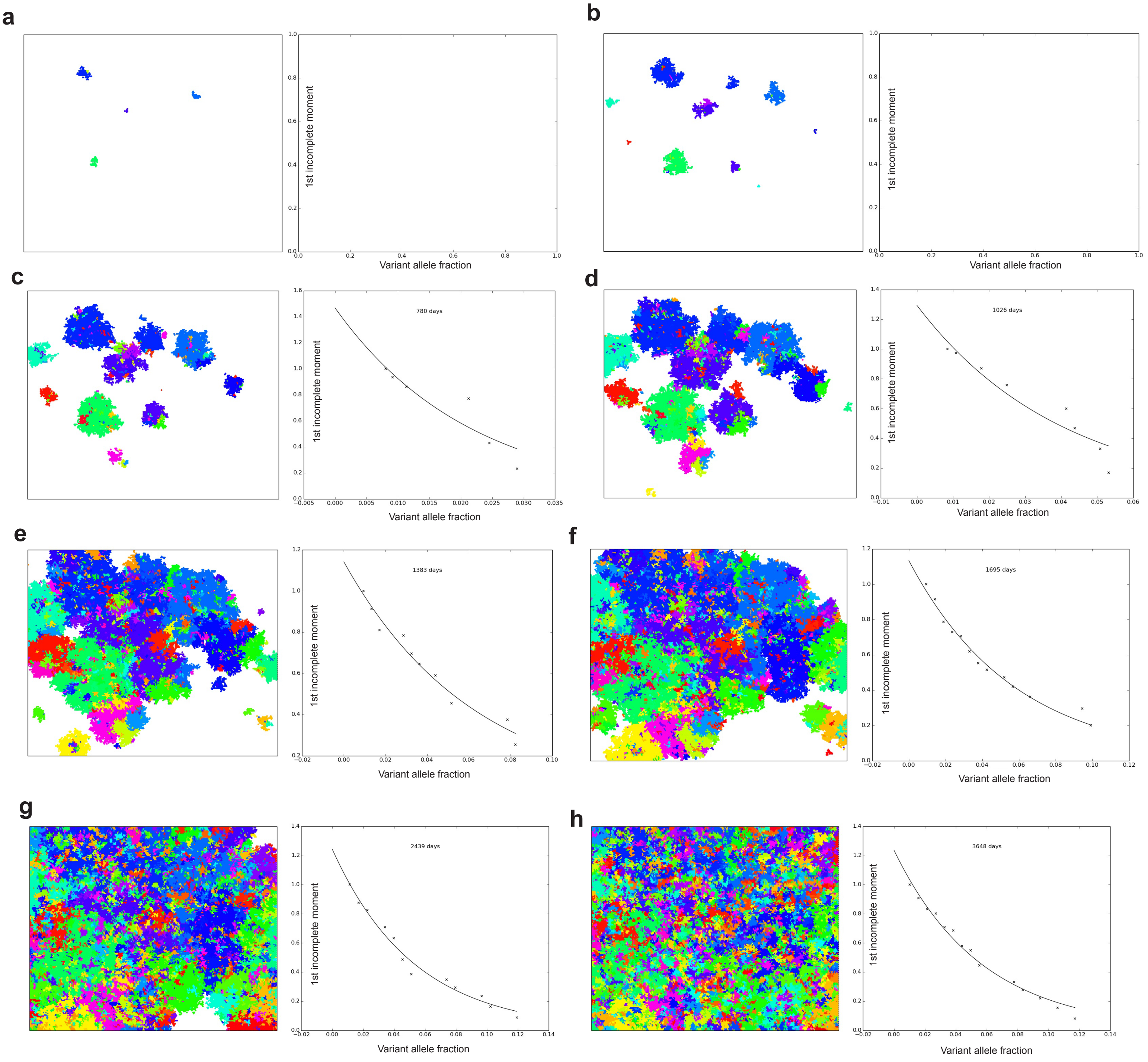

Supplementary Figure 9.
Visualisation of the effects of non-neutral mutation. Cells were simulated on a 200x200 hexagonal lattice with neutral mutation rate 10^{-2}, rate of stem cell loss/replacement is 0.5 $cell^{-1}week^{-1}$, non-neutral mutation rate 10^{-5} $cell^{-1}day^{-1}$. Wild type stem cells (white, not visible) stochastically acquire non-neutral mutations displayed as (arbitrarily assigned) coloured clones. The state of the lattice is illustrated at 228 days (a) 492 days (b) 780 days (c) 1026 days (d) 1383 days (e) 1695 days (f) 2439 days (g) and 3648 days (h).

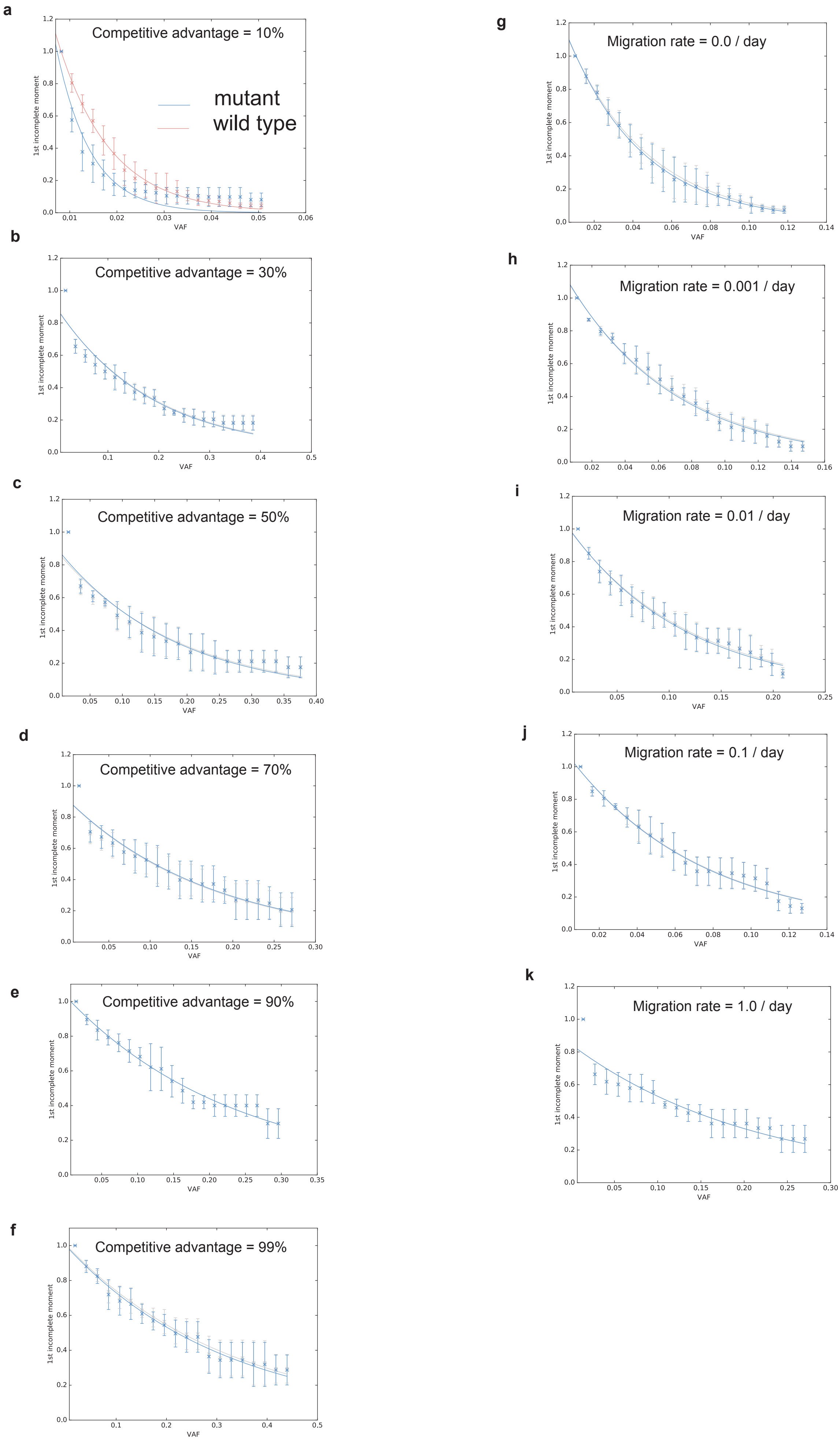

Supplementary Figure 10

Supplementary Figure 10.

Effects of magnitude of competitive advantage and rate of cellular migration on clone size distributions arising from clonal expansion of non-neutral mutations.

(a-f) Effects of magnitude of competitive advantage on clone size distributions. Cells were simulated on a 100$\times$100 lattice for a period equivalent to 10 years. Clones with VAF > 0.007 are plotted. Non-neutral mutations led to a reduction in the rate of loss of loss of the stem cell by (a) 10% (b) 30% (c) 50% (d) 70% (e) 90% and (f) 99%. Neutral mutation rate 10^{-3}cell^{-1}day^{-1}.

Each simulation was repeated 5 times and data is presented mean +/- S.D.

(g-k) Effects of rate of cellular migration on clone size distributions. The average rate of cellular migration in the lattice was varied from 0.0 cell^{-1}day^{-1} (g) 10^{-3} cell^{-1}day^{-1} (h) 10^{-2} cell^{-1}day^{-1} (i) 10^{-1} cell^{-1}day^{-1} (j) 1.0 cell^{-1}day^{-1} (k). Clones with VAF > 0.007 are plotted. Neutral mutation rate 10^{-3} cell^{-1}day^{-1}, non-neutral mutation rate 10^{-6}cell^{-1}day^{-1}. Each simulation was repeated 5 times and data is presented mean +/- S.D.

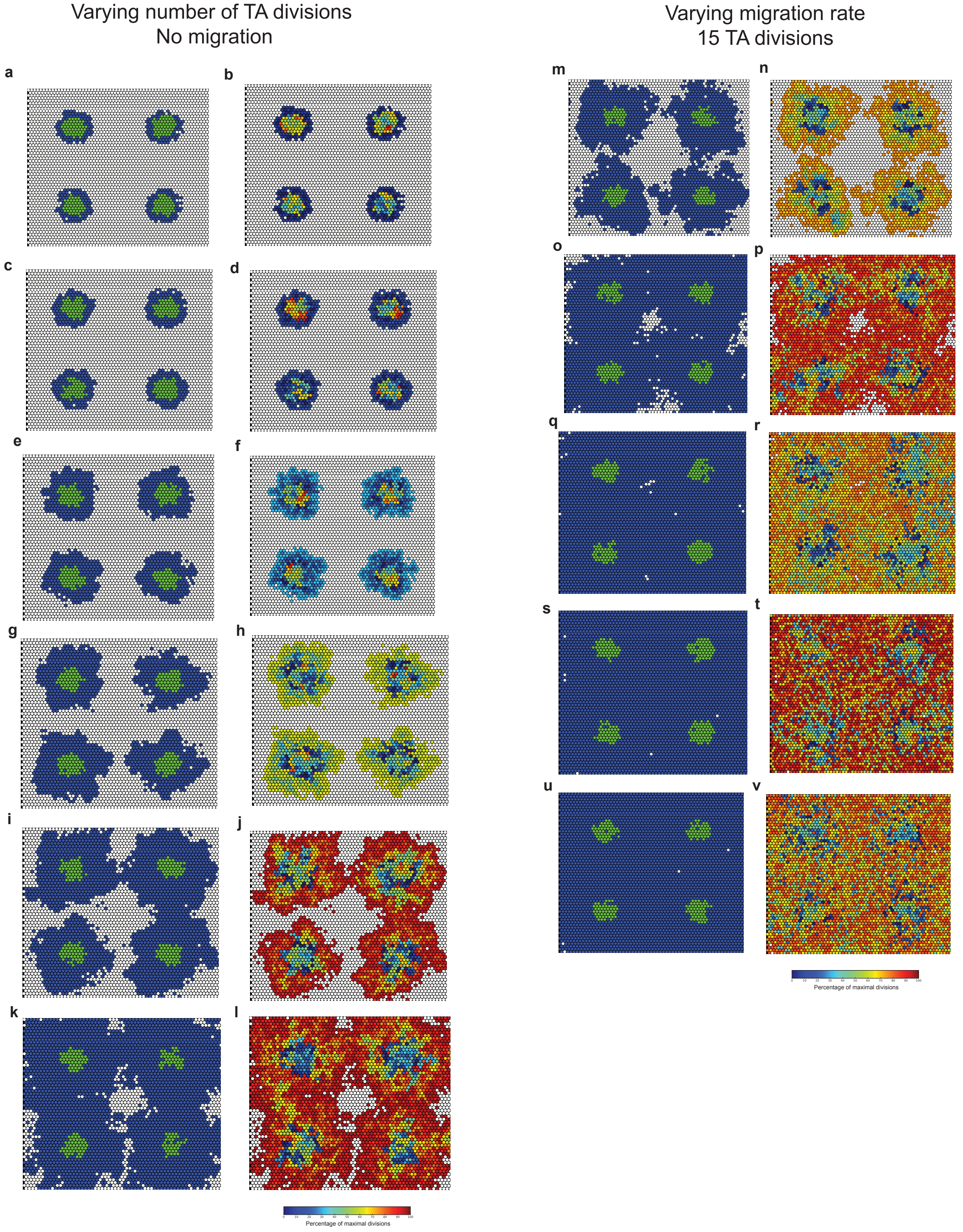

Supplementary Figure 11.
Simulation of stem cell clusters surrounded by transit amplifying cells on a hexagonal lattice. Clusters have average diameter of 10 cells and average separation of 20 cells. The lattice is illustrated in steady-state 100 days after initiation of the simulation. Either cellular identity (a,c,e,g,i,k,m,o,q,s,u; stem cells green, transit amplifying cells blue, empty lattice position white) or number of replications (b,d,f,h,j,l,n,p,r,t,v; blue low, red high) is indicated.
(a-l) Effects of number of transit amplifying (TA) cell divisions on lattice occupancy. The maximum number of TA divisions was varied from 1 (a,b), 2 (c,d), 5 (e,f), 10 (g,h), 15 (i,j), 20 (k,l).
(m-v) Effects of TA cell migration on lattice occupancy. Maximum TA cell divisions was fixed at 15. TA cell migration was varied from 0.0 cell^{-1}day^{-1} (m,n) 0.1 cell^{-1}day^{-1} (o,p) 0.5 cell^{-1}day^{-1} (q,r) 0.7 cell^{-1}day^{-1} (s,t) and 1.0 cell^{-1}day^{-1} (u,v).

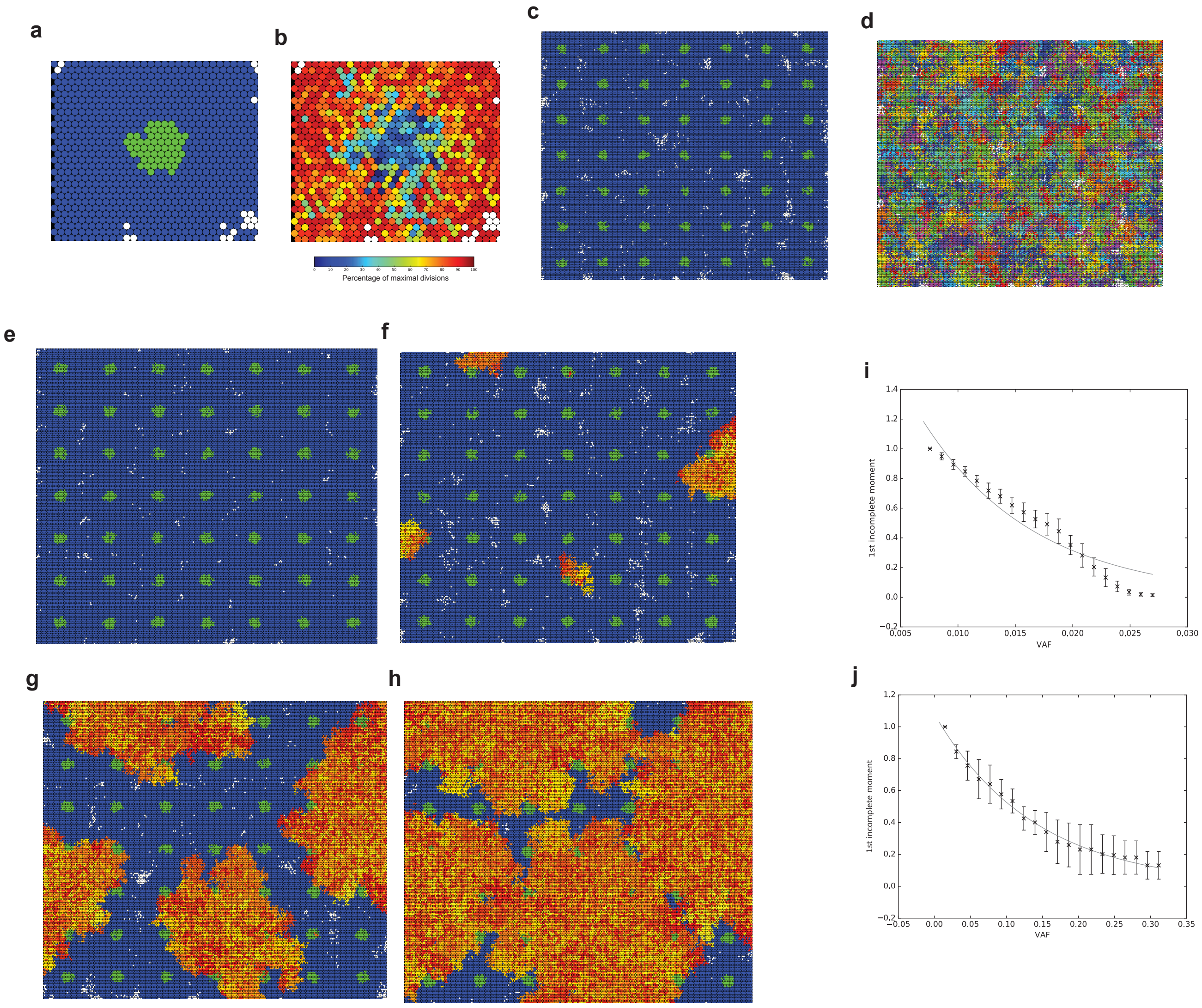

Supplementary Figure 12.
Effects of stem cell cluster size on clone size distributions. 49 stem cell clusters were simulated for 1000 days. Average stem cell cluster diameter was 10 cells with average cellular separation of 20 cells. Neutral mutation rate was 10^{-3} $cell^{-1}day^{-1}$ and non-neutral mutation rate was 10^{-6} $cell^{-1}$ day^{-1}.
(a-b) Visualisation of a single epidermal proliferating unit 100 days after initiation of the simulation. Cellular identity (a; green, stem; blue, TA; white, empty lattice space) and number of cellular divisions completed (blue, low; red, high) is indicated for a single proliferating unit.
(c) Cellular identity for 49 stem cell proliferating units 100 days after initiation of the simulation.
(d) Clonal identity (arbitrary color assigned to each clone) for 49 stem cell proliferating units 100 days after initiation of the simulation.
(e-h) Clonal expansion of non-neutral mutations (mutant stem cells, yellow-red; wild type stem cells, green; TA cells, blue; empty lattice spaces, white). 40 days (e) 220 days (f) 400 days (g) and 500 days (h) after initiation of the simulation.
(i) Clone size distribution for neutral mutations in the absence of non-neutral mutations.
(j) Clone size distribution for all mutations in the presence of non-neutral mutations. Clones with VAF > 0.007 are plotted. Each simulation was repeated 10 times and data is presented mean +/- S.D.

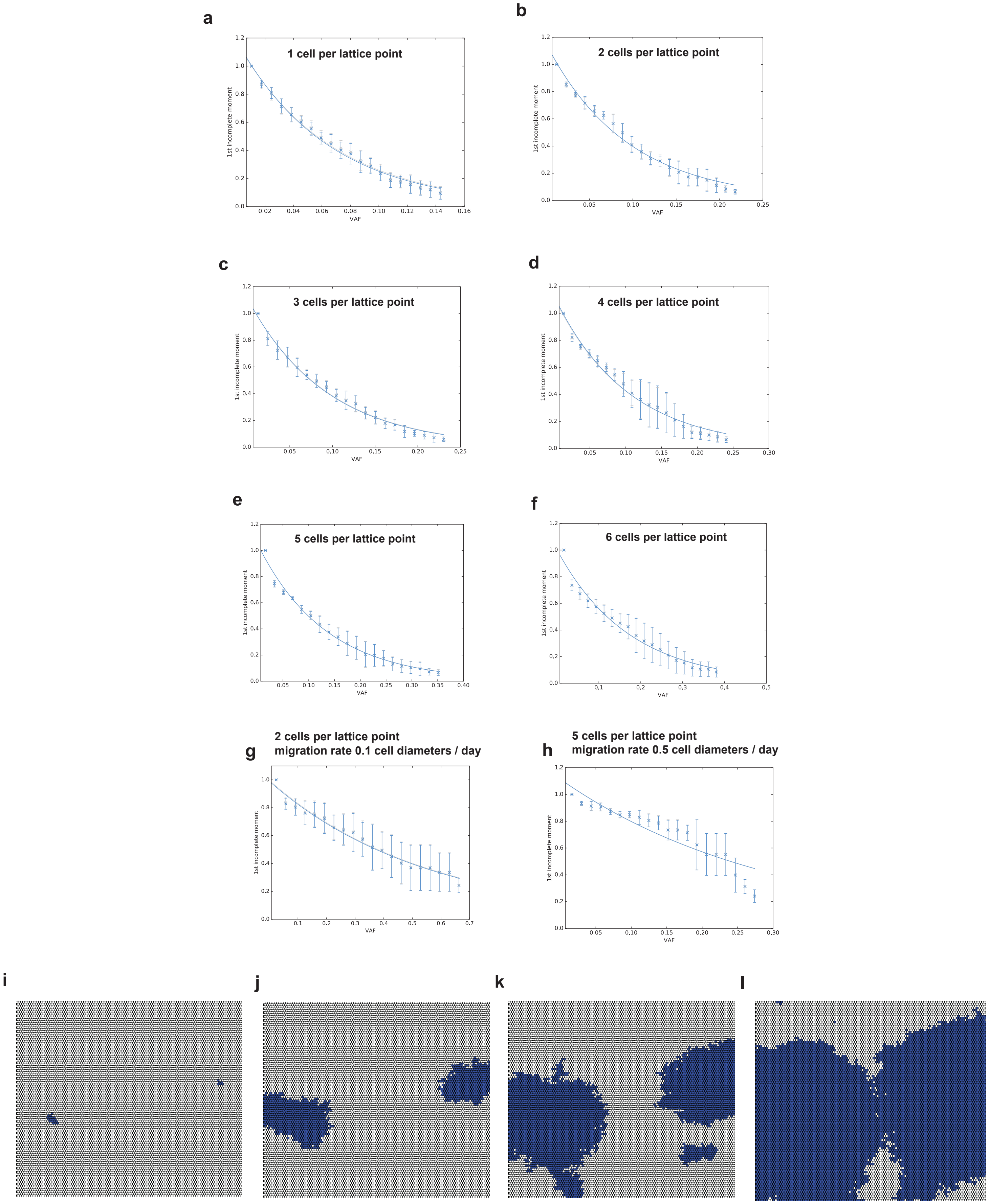

Supplementary Figure 13

Supplementary Figure 13.

Effects of number of cells per lattice point on clone size distributions arising from expansion of non-neutral mutations. Cells were simulated on a 200x200 lattice for a period equivalent to 10 years.

(a-f) 1 (a), 2 (b), 3 (c), 4 (d), 5 (e) or 6 (f) cells were permitted to occupy each lattice point. Each simulation was repeated 5 times and data is presented mean +/- S.D. Note differing scales on x axis.

Clones with VAF > 0.007 are plotted. Non-neutral mutations led to a reduction in the rate of loss of loss of the stem cell by 50%. Neutral mutation rate 10^{-3} $cell^{-1}day^{-1}$. Non-neutral mutation rate 10^{-6} $cell^{-1}day^{-1}$.

(g-h) Simulation of relaxation of spatial constraints in combination with cellular migration. Migration rate 0.1 $cells^{-1}day^{-1}$, 2 cells per lattice position (g), Migration rate 0.5 $cells^{-1}day^{-1}$, 5 cells per lattice position (h). Neutral mutation rate 10^{-3} $cell^{-1}day^{-1}$. Non-neutral mutation rate 10^{-6} $cell^{-1}$ day^{-1}. Simulation of 100x100 lattice for 1000 days, 5 repetitions data shown as mean +/- S.D.

(i-l) Spatial distribution of mutant clones when up to 5 cells are permitted to occupy the same lattice position. White indicates that all cells in a single lattice point are wild type stem cells. Blue indicates that at least one cell at that lattice position has a non-neutral mutation. The evolution of clones at 100 (i), 400 (j), 700 (k) and 1100 (l) days is indicated.

Supplementary References.

1. Simons, B. D. Deep sequencing as a probe of normal stem cell fate and preneoplasia in human epidermis. *Proc. Natl. Acad. Sci. U.S.A.* **113**, 128–133 (2016).

2. Klein, A. *The laws of cell fate in epidermal maintenance*. Ph.D. thesis, Cambridge University (2008).

3. Alcolea, M. P. *et al.* Differentiation imbalance in single oesophageal progenitor cells causes clonal immortalization and field change. *Nat. Cell Biol.* **16**, 615–622 (2014).

4. Lowell, S., Jones, P., Le Roux, I., Dunne, J. & Watt, F. M. Stimulation of human epidermal differentiation by delta-notch signalling at the boundaries of stem-cell clusters. *Curr. Biol.* **10**, 491–500 (2000).

5. Estrach, S., Legg, J. & Watt, F. M. Syntenin mediates Delta1-induced cohesiveness of epidermal stem cells in culture. *J. Cell. Sci.* **120**, 2944–2952 (2007).

6. Page, M. E., Lombard, P., Ng, F., Gottgens, B. & Jensen, K. B. The epidermis comprises autonomous compartments maintained by distinct stem cell populations. *Cell Stem Cell* **13**, 471–482 (2013).

7. Jensen, U. B., Lowell, S. & Watt, F. M. The spatial relationship between stem cells and their progeny in the basal layer of human epidermis: a new view based on whole-mount labelling and lineage analysis. *Development* **126**, 2409–2418 (1999).

8. Backvall, H., Wolf, O., Hermelin, H., Weitzberg, E. & Ponten, F. The density of epidermal p53 clones is higher adjacent to squamous cell carcinoma in comparison with basal cell carcinoma. *Br. J. Dermatol.* **150**, 259–266 (2004).

9. Sanchez-Danes, A. *et al.* Defining the clonal dynamics leading to mouse skin tumour initiation. *Nature* **536**, 298–303 (2016).

10. Watt, F. M. & Green, H. Involucrin synthesis is correlated with cell size in human epidermal cultures. *J. Cell Biol.* **90**, 738–742 (1981).

11. Jones, P. H. & Watt, F. M. Separation of human epidermal stem cells from transit amplifying cells on the basis of differences in integrin function and expression. *Cell* **73**, 713–724 (1993).
