# Supplementary material for: Spatial constraints govern competition of mutant clones in human epidermis
Source: Nat Commun. 2017 Oct 24;8:1119. doi: 10.1038/s41467-017-00993-8 (PMC5654977; doi:10.1038/s41467-017-00993-8)
Supplement: Supplementary file 3 — Description of Additional Supplementary Files [file 41467_2017_993_MOESM3_ESM.pdf]

Description of Additional Supplementary Files

File Name: Supplementary Movie 1
Description: 10 year simulation of clonal evolution on a 200x200 hexagonal lattice. Left panel: Wild type cells are evolving in neutral drift (white, not visible). Mutations arise sporadically causing a 2-fold reduction in rate of loss from the basal layer. Mutant cells are coloured. Colour is determined randomly according to the clonal identity. Neutral mutation rate is 10-3 , non-neutral mutation rate 10-6 cell-1 day-1 Right panel: Clone size distribution (all clones) is displayed for clones in excess of the size limit detectable by high throughput sequencing (VAF 0.007).

File Name: Supplementary Movie 2
Description: 10 year simulation of clonal evolution of stem cells (green), transit amplifying cells (TA; blue) and empty spaces (white) on a 315x315 hexagonal lattice. Stem cell cluster radius is 20 cells. Neutral mutation rate for all cells is 10-3 . Notch mutations (yellow-red) arise stochastically with rate 10-6 cell-1 day-1 and prevent differentiation of stem cells to TA cells. Unique clones within an expanding Notch-mutant clone are indicated by an arbitrary color within the yellow-red spectrum.

File Name: Supplementary Data 1
Description: Mutations identified by high throughput sequencing of human epidermis. Sample indicates the patient in which the mutation was identified. The genomic location of the mutation is specified by the chromosome, start and end columns. Sequence of the reference sequence (genomic control DNA for that individual) and the mutant sequence is indicated along with the gene in which the mutation is located. Variant allele fraction (VAF) and reads on forward and reverse strands are tabulated along with the total reads at that genomic location for that individual.
